# Coherence in the presence of absorption and heating in a molecule interferometer

**DOI:** 10.1038/ncomms8336

**Published:** 2015-06-11

**Authors:** J. P. Cotter, S. Eibenberger, L. Mairhofer, X. Cheng, P. Asenbaum, M. Arndt, K. Walter, S. Nimmrichter, K. Hornberger

**Affiliations:** 1 University of Vienna, Faculty of Physics, VCQ & QuNaBioS, Boltzmanngasse 5, A-1090 Vienna, Austria; 2 University of Duisburg-Essen, Faculty of Physics, Lotharstraße 1-21, 47048 Duisburg, Germany

## Abstract

Matter-wave interferometry can be used to probe the foundations of physics and to enable precise measurements of particle properties and fundamental constants. It relies on beam splitters that coherently divide the wave function. In atom interferometers, such elements are often realised using lasers by exploiting the dipole interaction or through photon absorption. It is intriguing to extend these ideas to complex molecules where the energy of an absorbed photon can rapidly be redistributed across many internal degrees of freedom. Here, we provide evidence that center-of-mass coherence can be maintained even when the internal energy and entropy of the interfering particle are substantially increased by absorption of photons from a standing light wave. Each photon correlates the molecular center-of-mass wave function with its internal temperature and splits it into a superposition with opposite momenta in addition to the beam-splitting action of the optical dipole potential.

## Abstract (web summary)

Extending matter-wave interferometry to nanoscale objects requires beam splitters that can cope with their internal complexity. Here, the authors demonstrate that the absorption of individual photons allows the center-of-mass coherence of large molecules to be maintained.

## Graphical abstract

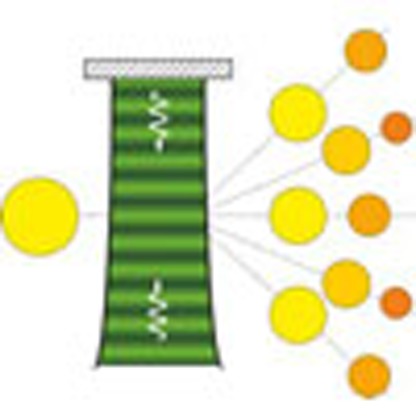

Quantum interferometry with large molecules and nanoparticles probes the limits of quantum physics^1^,^2^ and provides new methods to measure macromolecular properties^3^,^4^,^5^. It is therefore essential to understand the mechanisms underlying the coherent wave function division of nanoscale matter.

In atom optics, coherent beam splitters have been realised with material nanomasks^6^,^7^ as well as using standing light waves as phase^8^,^9^,^10^,^11^ or amplitude gratings^12^,^13^. They have been implemented using resonant laser light to drive coherent transitions between vibrational^14^, electronic^15^ or hyperfine^16^ states as well as off-resonant light in Bragg diffraction^17^ and Bloch oscillations^18^. In atom interferometry, the absorption of a photon can be followed by spontaneous emission into free space which can result in decoherence^19^,^20^,^21^,^22^.

Recently, the inherently rich internal structure of complex molecules has been exploited to achieve photo-depletion beam splitters using single-photon ionization^23^,^24^ and fragmentation^25^. Phase gratings have also been realised for molecules both in far-field diffraction^26^ and in Kapitza–Dirac–Talbot–Lau interferometry (KDTL interferometry)^27^. This has enabled quantum experiments with a wide variety of macromolecules—even using particles with a mass exceeding 10,000 a.m.u. (ref. 28).

Here, we demonstrate KDTL interferometry in a regime where a three-component beam-splitting mechanism occurs in a standing light wave. We observe matter-wave phase modulation induced by the electric dipole interaction between a polarizable particle and the laser field. In contrast to atoms, complex molecules can convert the energy of an absorbed photon into vibrational excitations, revealing no which-path information and enabling spatial coherence to be maintained. In addition, the absorption of photons induces an amplitude modulation of the matter wave in combination with an increase of the internal temperature of the molecule. This occurs preferentially when the molecule passes an antinode of the standing light wave. Because a molecule can only interfere with itself as long as all internal states are identical, only wave components of the same temperature class will interfere with each other.

## Results

### Beam splitting of complex molecules

**Figure 1:**
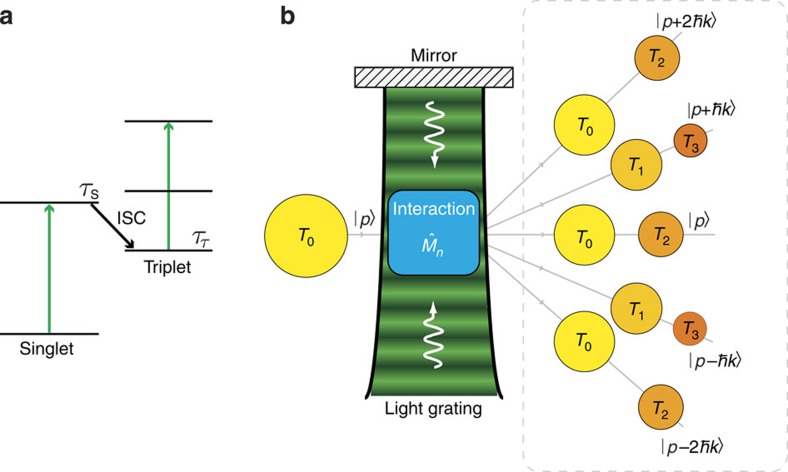
Coherent momentum transfer and heating in an optical grating. (**a**) Photoabsorption in the singlet ladder of C_70_ is followed by a rapid inter-system crossing (ISC) to the lowest triplet state^29^,^44^ whose lifetime exceeds 
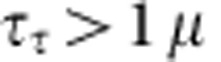
s, even at high temperatures^45^,^46^. The singlet-state lifetime is on the order of *τ*_s_≃1 ns. The absorbed photon energy is therefore predominantly dissipated non-radiatively into the vibrational degrees of freedom, increasing the internal temperature in steps of Δ*T* per photon. (**b**) A molecular ensemble characterized by the transverse momentum state |*p*〉 and internal, microcanonical temperature *T*_0_ diffracts at a light grating. The interaction of each molecule with the light grating, described by the operator 
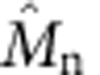
, correlates the internal temperature increase Δ*T* with a coherent momentum change Δ*p*=±*ħk*. An additional amplitude and phase modulation of the molecular wave function results in a superposition of diffraction orders, separated by two photon momenta for each temperature class. The temperature of each sub-ensemble is related to the number of absorbed photons *n* through *T*_*n*_=*T*_0_+*n*Δ*T*, where *T*_0_ is the internal temperature of the molecules when they reach the light grating.

To investigate the role of phase modulation, absorption-induced amplitude modulation and the transfer of optical coherence from the standing light wave to the interfering particle, we conducted a series of measurements using the fullerene C_70_. This particle was chosen because it is well characterized, it is prototypical for many more complex molecules and it is sufficiently large that it behaves as a heat sink for absorbed photons. Figure 1a shows its lowest electronic energy levels^29^. It has a strong inter-system crossing, which results in fast internal thermalization after a photon has been absorbed. In ∼1 ns, the population of the excited singlet state is transferred to the lower lying triplet, with a probability exceeding 90%. Figure 1b shows how the three beam-splitting processes combine for a spatially coherent molecular beam to produce interferograms, which are associated with different internal temperatures and yet still contribute to the same molecular density pattern. The phase and measurement-induced gratings contribute a momentum superposition in multiples of 2*ℏk*, whereas single-photon absorption contributes coherently to the momentum change in multiples of *ℏk*. Here, *ℏ* is the reduced Planck constant and *k* is the wavenumber of the laser.

### Experimental set-up

**Figure 2:**
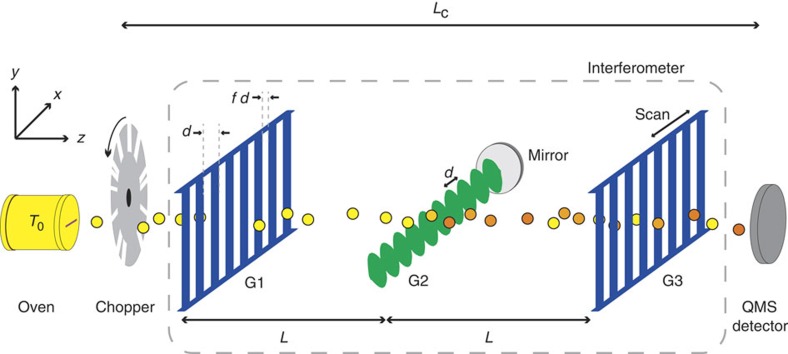
Time-resolved measurement in the KDTL interferometer (KTDLI). An effusive molecular beam leaves an oven with temperature *T*_0_. Cross correlating a pseudo-random sequence of openings in a chopper wheel with the arrival time of molecules at the detector provides time-of-flight information, and therefore velocity resolution. The KDTLI is a three grating, near-field matter-wave interferometer. G1 and G3 are material gratings, whereas G2 is formed from a standing light wave. Adjacent gratings are separated by a distance of *L*. All three have a period *d*=266 nm and the material gratings have an opening fraction of *f*∼0.42. Sinusoidal interference patterns in the molecular density distribution are revealed by translating G3 laterally in front of a quadrupole mass spectrometer (QMS) positioned at a distance *L*_C_ after the chopper wheel. Some particles absorb one or more photons in the light grating resulting in an internal temperature increase, illustrated here by a change in colour.

A sketch of the KDTL interferometer is shown in Fig. 2. Molecules are sublimed in an oven at a temperature of *T*_0_≃950 K to form an effusive beam along *z*. Before leaving, the molecules reach thermal equilibrium with the oven and therefore arrive at the light grating with an internal temperature *T*_0_. As the beam propagates, it encounters three gratings in a KDTL^27^ configuration. Here, two material gratings are separated by a distance 2*L*, with an optical grating centred between them. The material gratings G1 and G3 are made from silicon nitride with a period of *d*≃266 nm and an opening fraction of *f*=0.42≃110 nm/d. By virtue of Heisenberg's uncertainty relation, the narrow spatial confinement provided by G1 causes the molecular wave function to expand^30^. A beam of C_70_ molecules with velocities ∼180 ms^−1^ coherently illuminates several periods of the second grating, which places the experiment in the Kapitza–Dirac regime. The standing light wave, G2, is formed by retro-reflecting a Gaussian laser beam from a plane mirror several hundred micrometres away from the molecular beam. The laser beam has a wavelength of *λ*=2*π*/*k*=2*d*≃532 nm and is focussed to waists of *w*_z_≃20 μm and *w*_y_= 425 μm on the mirror. The short transition time through G2 ensures that the molecular position remains unchanged inside the grating even though it receives a superposition of momentum kicks (Raman–Nath regime)^31^.

Quantum interference produces a sinusoidal particle density at the position of G3 if *L*=105 mm is close to a multiple of the Talbot length *L*_T_=*mv*_z_*d*^2^/2*πℏ*. Here, *m* is the mass and *v*_z_ the forward velocity of the molecule. The detailed shape of the interference pattern depends on the opening fraction and period of the material gratings as well as on the intensity of the standing light wave. Particles are detected using an electron-impact ionization quadrupole mass spectrometer (QMS). Both the fringe pattern and G3 have the same period, allowing interference to be measured by translating G3 along *x*. For all practical situations, the resulting interference patterns are well represented by a sinusoidal function^32^ characterized by the visibility *V*=*A*/*μ*, where *μ* and *A* are the mean number of counts and the amplitude, respectively.

### Time-resolved interferometry

Accurate knowledge of the molecular velocity is required to observe the three beam-splitting mechanisms under investigation.

**Figure 3:**
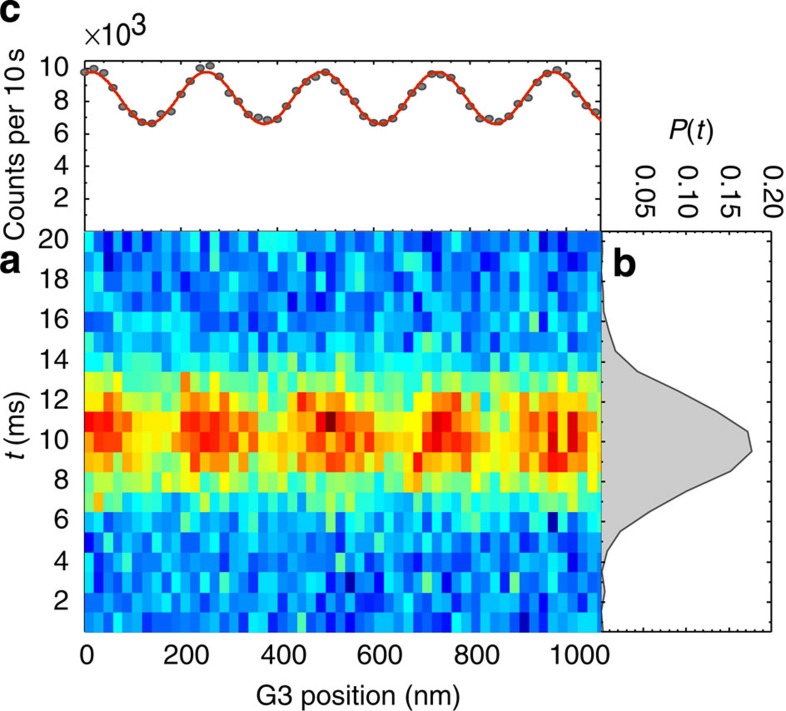
Time-of-flight interferometry. (**a**) Time-of-flight distributions were recorded for a number of different lateral positions of grating G3. For this image, a time resolution of 1 ms was used. (**b**) The time-of-flight probability density obtained by summing over the positions of G3. It is well described by a Gaussian with a mean of 9.7 ms and a standard deviation of 2.1 ms. (**c**) Interference pattern obtained by summing over all time-of-flights.

This is achieved by chopping the molecular beam in a pseudo-random sequence^33^,^34^ using a slotted rotating disc with a series of appropriate openings, positioned at *L*_C_=1.74 m from the QMS. The time of flight and velocity distribution can then be obtained by deconvolving the arrival times with the measured chopper transmission sequence. More details can be found in the Methods. Close to the mean time of flight, we have improved the molecular velocity resolution by an order of magnitude over previous experiments^32^. Figure 3a shows a molecular interferogram obtained for a light grating power of 3.5 W. This is constructed by recording molecular time-of-flight distributions for different positions of G3. Figure 3b,c show the corresponding time-of-flight distribution and velocity averaged interference pattern, produced by summing over all grating positions and time-bins, respectively. The velocity-selected patterns are required to observe all three diffraction mechanisms, while the integrated curve is used to determine the overall fringe phase (see Methods).

**Figure 4:**
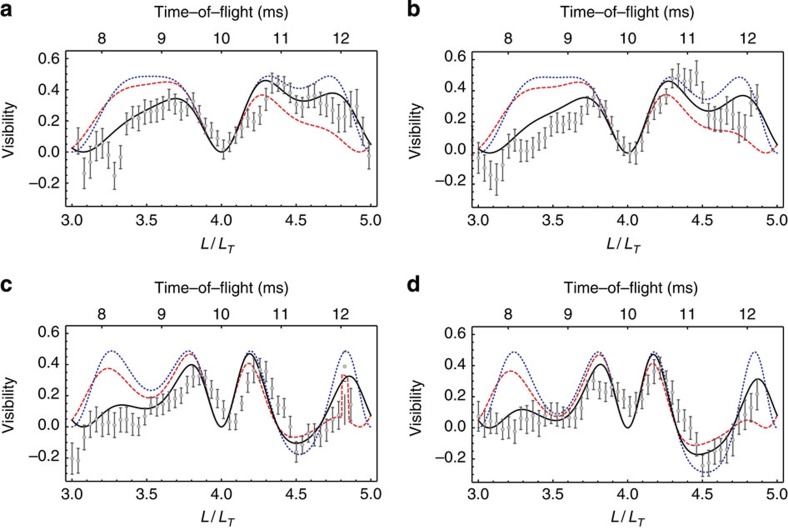
Visibility as a function of Talbot order. Grey data points show the fitted sinusoidal interference visibility, 
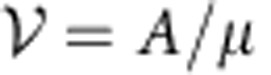
, where *A* and *μ* are the sinusoidal amplitude and mean number of counts. Error bars are the 1*σ* uncertainties extracted from the fit. The panels correspond to a laser power of (**a**) *P*=3.50 W, (**b**) *P*=3.75 W, (**c**) *P*=5.25 W, (**d**) *P*=5.75 W. The dotted blue line shows the quantum prediction of a pure phase grating. The dashed red line includes photon absorption as a random walk^32^. The solid black line shows our quantum model from equation (5), which includes the interplay of coherent absorption and phase modulation. Negative visibilities arise when the phase of the interference pattern of a given time bin is shifted by *π* with respect to the phase of the velocity averaged signal. This only occurs if some fraction of the ensemble absorbs an odd number of photons.

Figure 4 shows the measured time-resolved sinusoidal visibility as a function of Talbot order *L*/*L*_T_. We compare it with the model of a pure phase grating (blue, dotted), a phase grating modified by photon absorption as a random walk^32^ (red, dashed) and the full quantum model (black, solid) developed in the following section. We find good agreement between the full quantum model and our data, once a constant visibility reduction factor of 0.93(3), has been included. See Methods for more details. In contrast, the visibility for the random walk model of absorption is systematically higher than the data between odd and even Talbot orders and lower between even and odd. The phase grating description tends to overestimate the visibility for all Talbot orders. Only the coherent quantum model provides good agreement for all Talbot orders.

### Theoretical model

The electric field of a non-resonant standing light wave interacts with the molecular polarizability and creates a sinusoidal dipole potential. This imprints a phase onto the molecular wave function that varies between zero at the grating nodes and *ϕ*_0_ at its antinodes. If the molecular absorption cross-section is equal to zero, the laser acts as a pure phase grating, causing a plane molecular wave to split into a coherent superposition of momenta, separated by integer multiples of 2*ℏk*. This transforms the initial momentum state |*p*〉 into




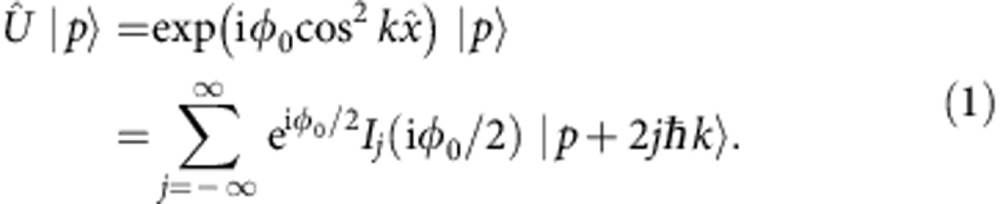




Here, 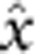 is the position operator. The relative weight of the *j*-th diffraction order is proportional to a modified Bessel function *I*_*j*_. This can be controlled by tuning the strength of the phase modulation *ϕ*_0_, which is proportional to the laser power as well as the particle’s optical polarizability and transit time through the grating.

In the presence of absorption, the situation is different. Each absorbed photon adds ±*ℏk* to the molecular momentum and increases the internal energy of the molecule by *ℏkc*, where *c* is the speed of light. The absorption thus correlates the internal state of the molecule with its centre-of-mass motion. For each sub-ensemble with a fixed number of absorbed photons *n* and increased internal temperature *T*_*n*_, the standing light wave acts both as a phase grating and an amplitude grating. If a molecule with a non-zero absorption cross-section diffracts without actually absorbing a photon, *n*=0, it is more likely to have passed at a position *x* close to a node of the standing wave. The probability for that process is 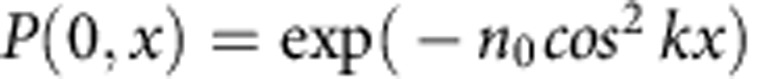, where *n*_0_ is the average number of photons absorbed at an antinode, see equation (6) in the Methods. According to the quantum measurement postulate, the conditional transformation of the momentum eigenstate |*p*〉 is therefore




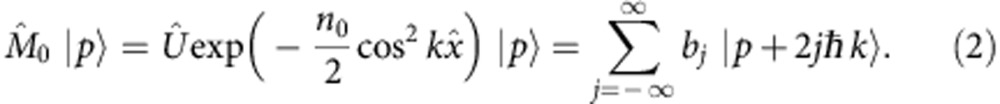




Here, 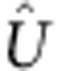 is the unitary transformation from equation (1), imposed by the phase grating. The exponential term in equation (2) modulates the amplitude such that the transmission probability 
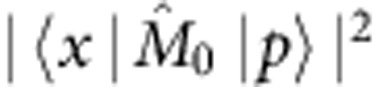
 amounts to the Poissonian probability *P*(0, *x*) for no absorption. Even though no photon is absorbed, diffraction still results from a combination of phase and amplitude modulation of the wave function. The grating coefficients *b*_*j*_=exp(i*ϕ*_0_/2−*n*_0_/4) *I*_*j*_ (i*ϕ*_0_/2−*n*_0_/4) contain both phase and absorptive components, which describe the relative amplitudes and phases of the diffraction orders.

The formalism of equation (2) can also be used to describe the effect of single-photon ionization or fragmentation gratings when only intact neutral particles, that is, those with *n*=0, are transmitted to the detector^24^,^25^. In contrast, the absorption of green photons in KDTL interferometry here is non-depleting, that is, all molecules contribute to the final pattern, independent of the number of photons they have absorbed. Therefore, all sub-ensembles with *n*>0 must be taken into account. In particular, the probability for absorbing exactly one photon is 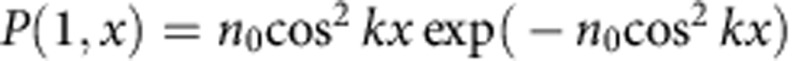 and the conditional diffraction of molecules labelled by *n*=1 is




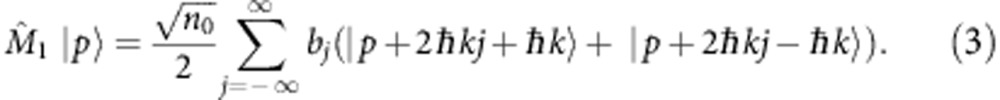




Here, 
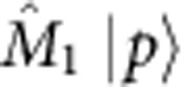
 describes a coherent amplitude and phase modulation, but with an additional coherent splitting of the wave function by one photon recoil. Because the standing light wave is a superposition of two counter-propagating running waves, each absorbed photon is in a superposition of two anti-parallel directions, which are imprinted onto the molecular momentum. The general transformation for *n* absorbed photons is then




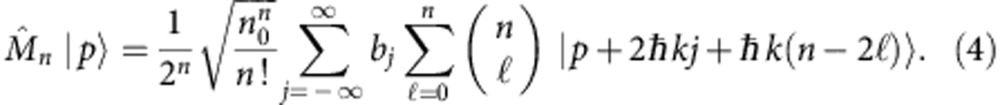




It includes the effects of coherent phase modulation, absorption-induced amplitude modulation and coherent recoil splitting. The latter is represented in equation (4) by the binomial sum over all combinations of *n* single-photon recoils, which result in a net recoil between ±*nℏk*. Absorption of *n* photons by any individual C_70_ molecule leads to a step-wise heating, predominantly by populating its 204 different vibrational degrees of freedom. The resulting interference pattern can therefore be assigned to a distinct internal state at the microcanonical temperature *T*_*n*_=*T*_0_+*n*Δ*T*. For a constant heat capacity^35^, *C*_v_=202*k*_*B*_, the temperature increase amounts to *n*Δ*T*=*nℏkc*/*C*_v_=*n*·134 K.

**Figure 5:**
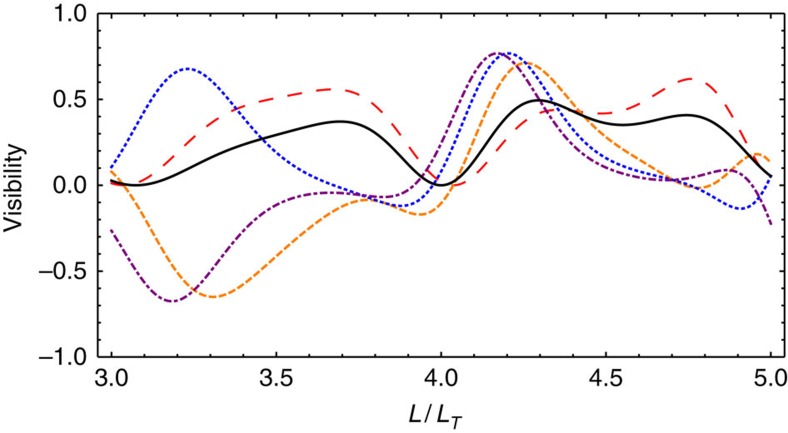
Conditional fringe visibilities as a function of Talbot order *L*/*L*_T_. The parameters are the same as in Fig. 4a. The long-dashed red line shows the visibility for molecules that passed the laser grating without absorbing. The dashed-orange, dotted blue and dash-dotted purple lines show the visibility for the case of one, two and three absorbed photons, respectively. Negative visibilities correspond to fringe patterns with a phase shifted by *π*. The solid black line shows the incoherent sum of all interferograms associated with the different absorption orders, as given by equation (5).

Different internal temperature classes of the same molecule cannot coherently contribute to the same interference pattern, since they are distinguishable. Figure 5 shows the visibility corresponding to different ‘thermally labelled’ ensembles *n*=0,1,2,3 and the incoherent sum of all interferograms. In principle, a molecule detector sensitive to the internal energy of the particle would be able to resolve these. However, our electron-impact ionization detector does not distinguish between internal states. The total fringe pattern is therefore an incoherent sum of all conditional interferograms, each determined by the state transformation of equation (4) for a given *n*. An ensemble of molecules in a mixture of internal states can still produce high contrast interference patterns. The sinusoidal visibility which characterizes the interference pattern for an incoherent mixture of internal states is,




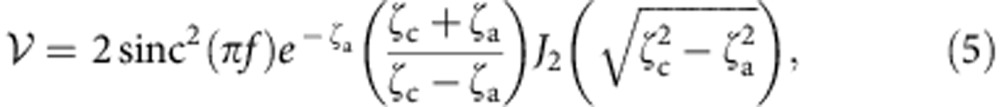




with *J*_2_ the second order Bessel function. Here, *ζ*_a_=*n*_0_(1−cos(*πL*/*L*_T_))/2 and *ζ*_c_=*ϕ*_0_ sin(*πL*/*L*_T_) describe the effects of photon absorption and phase modulation, respectively. Equation (5) differs from a random walk model of absorption^32^ only in the sign of *ζ*_c_. This is a direct consequence of the absorbed photon being in a superposition of momentum states.

We have assumed that the absorption cross-section is independent of the internal state and that the photon energy is not re-emitted^35^. Although these approximations are justified here, the storage of many photons would eventually lead to the emission of thermal radiation and decoherence^36^ for 
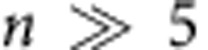
. In our present experiments, ∼1% of all molecules absorb this much in the antinodes.

## Discussion

We have shown that three intricate processes combine in the diffraction of nanoscale matter at a standing light wave if the absorbed photon is swallowed and converted internally into heat. We find that to correctly describe molecular diffraction at a light grating, phase- and absorption-induced amplitude modulation must be combined with a coherent recoil splitting. The internal degrees of freedom can be modelled as a reservoir whose microcanonical temperature reflects the number of absorbed photons.

A velocity resolution much better than one Talbot order is required to observe the difference between the full quantum model and a random walk description of absorption. We also note that in far-field diffraction both models give the same predictions. The difference can therefore only be observed in near-field matter-wave interferometry and only if the grating transformation involves a finite phase modulation *ϕ*_0_.

We note that the absorption mechanism, which creates the momentum coherence is conceptually similar to that of an atom spontaneously emitting a photon close to a mirror^37^. In both cases, the mirror erases information about the photon’s direction of propagation.

Multi-photon absorption in a standing light wave is closely related to the physics of quantum random walks^38^. In contrast to classical walks where each recoil only opens one more path in a system of binary decisions—such as the evolution of trajectories in a Galton board—quantum walks exploit the power of superposition. This is similar to the probability distribution functions found in recent quantum walk experiments^39^,^40^.

Single-photon absorption in a running wave is the mechanism underlying the beam-splitting process in Ramsey–Bordé interferometry^15^. It correlates momentum and internal state in a reversible way that allows one to close the interferometer using absorption followed by stimulated emission. Macromolecules at an absorbing non-depleting light grating are different in that all energy is converted into vibrational excitations. In contrast to Ramsey–Bordé interferometry, the entanglement between the internal energy and the molecular momentum persists and cannot be undone by a second photon. One might hope to one day measure this correlation using temperature resolving superconducting stripline detectors^41^.

The concept of thermal labelling is unique to macromolecule interferometry and potentially useful for realising measurement-induced diffraction gratings for particles that neither ionize, fragment nor re-radiate upon absorption. Talbot–Lau interferometry with a spatially incoherent source^42^,^43^ requires an ‘absorptive’ beam splitter, at least in the first grating. However, this does not necessarily imply a physical removal of particles. One can conceive a temperature-labelling beam splitter, which depletes a certain temperature class by heating the molecule in the grating's antinode without removing the particle physically from the beam. Again, this can be realized once detectors become available that can discriminate different internal molecular energies.

## Methods

### Time-of-flight measurements

Time-of-flight distributions are recorded for different positions of G3. The molecular count rate is measured in bins of 100 μs and convoluted with the chopper sequence to obtain the time-of-flight distribution^33^,^34^. The disc is partitioned into 255 transmissive or blocking segments with 50% molecular throughput and rotates with 16.5 Hz. The time-of-flight distributions have a mean of *μ*_t_=9.7 ms and s.d. of *σ*_t_=2.1 ms. The distance between the chopper and the QMS is *L*_C_=1.74 m, resulting in a velocity distribution centred ∼180 ms^−1^ with a full-width half-maximum of 45 ms^−1^. For a mean time of flight of 9.7 ms, the velocity resolution of 2% represents an order of magnitude improvement compared with our previous experiments.

### Absorption in the light grating

The standing-wave intensity distribution determines the position-dependent Poissonian probability *P*(*n*, *x*) for a particle to absorb *n* photons,




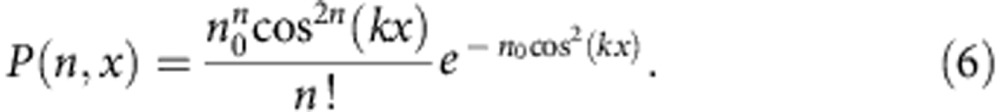




Here, *n*_0_=2*σε*_0_*ϕ*_0_/*kα* is the mean number of photons absorbed in the antinode of the light field, *P* is the running wave power of the laser, *σ* is the optical absorption cross-section and *α* is the optical polarizability. The phase modulation parameter, 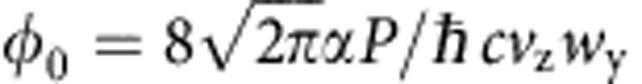, is proportional to the optical polarizability *α*. Both *σ* and *α* at 532 nm have been measured previously^5^,^32^, *σ*=1.97(6) × 10^−21^ m^2^ and *α*=4*πε*_0_ × 114(13) Å^3^.

The light grating has a waist of 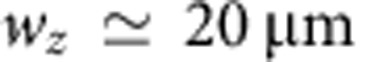 along the axis of the molecular beam. A particle with velocity *v*_*z*_ takes a time 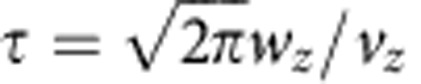 to traverse the light grating. Here, particle velocities are typically in the range *v*_*z*_=135–225 ms^−1^, resulting in interaction times of hundreds of nanoseconds. Such short times place the molecule-grating interaction in the Raman–Nath regime where their transverse motion can be neglected. The reduced centre-of-mass density matrix of the incident molecule ensemble, *ρ*, then evolves according to the master equation,




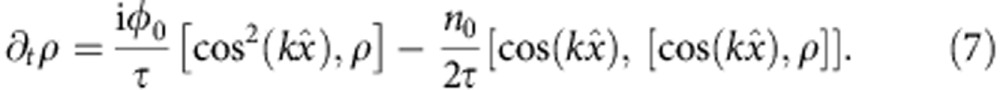




Here, the first term describes the coherent phase modulation of the molecular ensemble through the dipole interaction with the laser field. The second term represents the net effect of absorption after tracing over the reservoir of internal degrees of freedom, assuming a constant absorption cross-section. This simplifying assumption is justified for the present experiment, since 
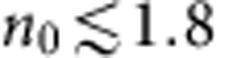
. Integrated over time, the overall transformation of the centre-of-mass state at the standing-wave grating can then be written as,




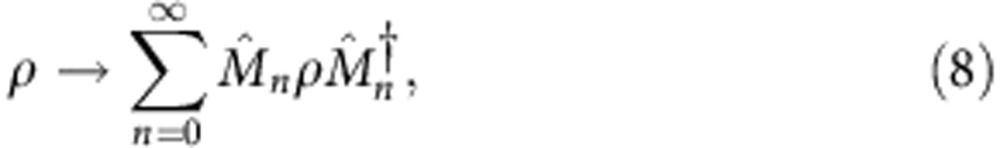




which is an incoherent sum of the conditional diffraction terms discussed in the main text. The expression for the visibility presented in equation (5) then follows directly by incorporating this transformation into the theoretical framework used to describe KDTL interferometry, presented previously^32^.

### Extracting visibilities

The phase and period, *θ* and *X*, were obtained for each interferogram by fitting *A* cos(2*πx*/*X*+*θ*)+*μ* to the velocity averaged interference pattern of each interferogram. These were obtained by summing over all time of flights between 5.7 and 13.7 ms. This range contains roughly 95% of the molecules arriving at the detector. The visibility, 
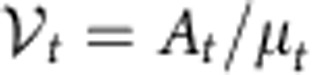
, was then determined for each time-bin of the interferogram by fitting the function *A*_*t*_ cos(2*πx*/*X*+*θ*)+*μ*_*t*_, where *A*_*t*_ and *μ*_*t*_ are the amplitude and mean number of counts for time bin *t* of the interferogram. This ensures that the phase and frequency of the time dependent interference patterns are constant throughout and reduces the number of free parameters in the visibility fits to only two. Error bars are the 1*σ* uncertainties returned by the fits.

### Visibility reduction

The theory curves in Fig. 4 can be plotted using no free parameters as all terms in equation 5 are known. However, we found that a constant visibility reduction factor of 0.93(3) was required to find good agreement between the theoretical predictions and our data. A change in *σ* or *α* cannot explain this reduction. The maximum visibility obtained for a given molecule is partially determined by the alignment of the interferometer. For example, a roll angle in G2 of ∼0.1 mrad with respect to G1 or G3 is sufficient to explain this. The same scaling factor was applied to the random walk model for absorption and phase grating description also plotted in Fig. 4.

## Additional information

**How to cite this article:** Cotter, J. P. *et al*. Coherence in the presence of absorption and heating in a molecule interferometer. *Nat. Commun.* 6:7336 doi: 10.1038/ncomms8336 (2015).
