# Erratum: Coherence in the presence of absorption and heating in a molecule interferometer

**DOI:** 10.1038/ncomms10121

**Published:** 2015-11-27

**Authors:** J. P. Cotter, S. Eibenberger, L. Mairhofer, X. Cheng, P. Asenbaum, M. Arndt, K. Walter, S. Nimmrichter, K. Hornberger

Nature Communications 6: Article number: 7336 10.1038/ncomms8336 (2015); Published: 06112015; Updated: 11272015

In Fig. 4c of this article, the second-last data point and the red-dashed fit were accidentally altered during the production process. The correct version of the figure appears below.


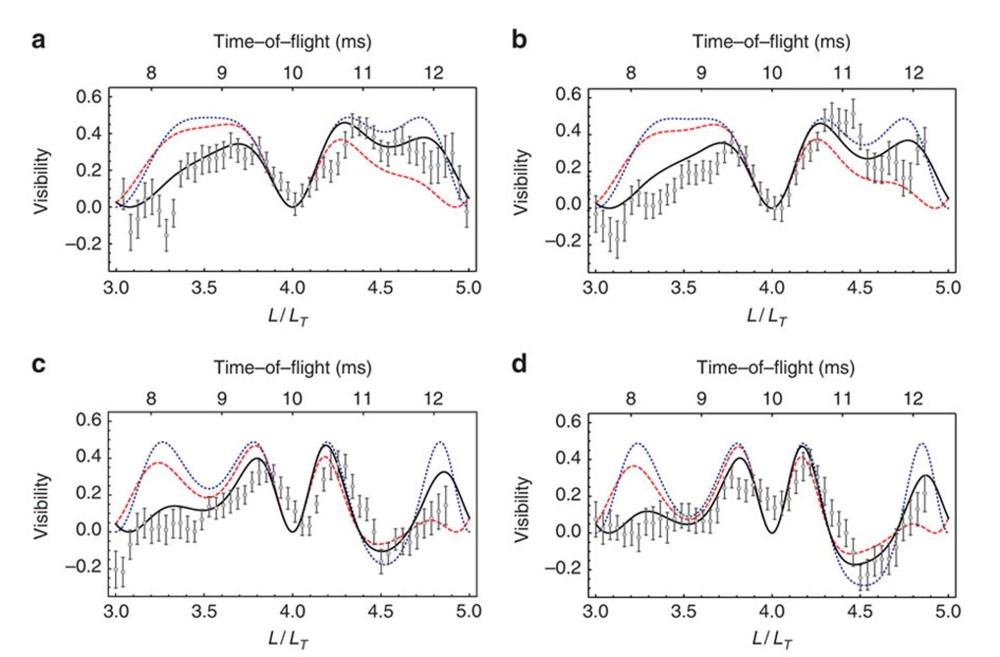


**Figure 4**

